# The measurement of microplastics in surface water and their impact on histopathological structures in wading birds of district Lahore

**DOI:** 10.3389/ftox.2024.1484724

**Published:** 2025-01-15

**Authors:** Shaza Mehboob, Khalid Mahmood Anjum, Hamda Azmat, Muhammad Imran

**Affiliations:** ^1^ Department of Wildlife and Ecology, University of Veterinary and Animal Sciences, Lahore, Pakistan; ^2^ Department of Fisheries and Aquaculture, University of Veterinary and Animal Sciences, Lahore, Pakistan; ^3^ Institute of Biochemistry and Biotechnology, University of Veterinary and Animal Sciences, Lahore, Pakistan

**Keywords:** microplastics, surface water pollution, metropolitan city Pakistan, tissue damage, red wettled lapwing

## Abstract

Plastics are globally considered a significant threat, particularly to metropolitan areas, due to the extensive use of plastic products. This research is the first of its kind to document microplastics contamination and its effects on Red wettled lapwing (Vanellus indicus). The concentration of microplastics (MPs) was measured from surface water at different locations including canals and drains, which are the primary sources of MPs pollution in the metropolitan city Lahore, Pakistan. The highest MPs concentration was recorded in the main stream of the Ravi River, with an average concentration of 5,150 ± 7.5 particles/m^3^. In addition, considering the different shapes of MPs, fibers were found to be most abundant at Site I (Main Stream of River Ravi), with the highest mean concentration of 92.4 ± 0.3 particles/m^3^, whereas the lowest mean concentration of 29.9 ± 0.1 particles/m^3^ was observed. In contrast, fragments were predominant at Site II (Shahdara Drain), with the highest and lowest mean concentrations of 42.6 ± 0.3 and 21.7 ± 0.1particles/m^3^, respectively. Chemical analysis revealed that most fragments, fibers; and beads belonged to the polyethylene class, while sheets were categorized as polypropylene and foam as polystyrene. The large MPs with particle size ranging from 400 μm to 5 mm were most abundant at both locations. Particles smaller than 0.5 mm were the most prevalent (56%) at Site I, while Site II showed the lowest proportions for size ranges 0.5–1 mm (24%), 1–2 mm (16%), 2–3 mm (8%), 3–4 mm (5%), and 4–5 mm (3%). The frequency of occurrence (%FO; prevalence) of plastics in necropsied birds was 89.7%. A total of 120 items were analyzed: 64 fibers, 23 fragments, 10 pieces of foam, 14 pieces of sheet, and 9 beads. Of the total ingested plastic debris analyzed, the largest proportion was comprised of polyethylene, making up 46% of the samples. Birds from Site I (Main Stream of River Ravi) had 100% of their organs containing plastic items compared to those from Site II (Shahdara Drain). Quantitative and qualitative histopathological analyses were performed to examine variations in prevalence percentage, frequency, and histological alteration indices (HAI) as a consequence of MPs exposure on the health of wild species. Tissue samples from the liver and kidneys of the Red-wattled lapwing were analyzed, and comparisons were made to assess the extent of damage and degree of alteration in bird organs. The study evaluated the impacts of ingested MPs, which induced inflammatory and anatomical responses in *V. indicus*. Significant tissue damage was observed, including considerable inflammatory responses, evident cellular swelling in many renal tubular epithelial cells, and pyknotic nuclei, which were major causes of necrosis and apoptosis. Prevalence percentage and frequency were significantly higher at Site I compared to Site II. The highest prevalence percentages in the liver and kidneys were 90% and 85%, respectively, manifesting as degeneration of hepatocytes and necrosis in renal tubular epithelial cells in response to 0.5–1 mm sized MPs. The lowest prevalence percentage, 5%, was observed as congestion of sinusoids and hyperemia in response to 4–5 mm sized MPs. The frequency and prevalence percentages followed the order: 0.5 mm > 0.5–1 mm > 1–2 mm > 2–3 mm > 3–4 mm > 4–5 mm > 0 mm (0 mm as control).This investigation contributes to the growing documentation of MPs abundance in freshwater ecosystems and provides a baseline for future studies on MPs pollution in the Ravi River.

## 1 Introduction

Synthetic materials of the modern era, defined by the European Chemical Agency (ECA) as particles with less than 5 mm diameter, are considered microplastics (European Chemical Agency, 2020). These particles vary in size and morphology (Duis and Coors, 2016) and are produced intentionally and artificially for consumer products, such as microbeads found in cosmetics, facial scrubs, detergents, sunscreens, and drug delivery systems, are classified as primary microplastics (Lambert and Wanger, 2018). Secondary microplastics are formed after degradation of large plastics and plastic wares into smaller fragments due to processes like UV photodegradation, biodegradation, sand and water abrasion, temperature changes, and hydrolysis (Chubarenko et al., 2020). UV photodegradation and weathering processes cause the breakage of chemical bonds in plastic polymers, leading to their fragmentation (Susanti et al., 2020; Casillas et al., 2023).

Global plastics production has dramatically increased over the last few decades (Microplastics, 2020), contributing to the build-up of plastic debris at an elevated level (Wong et al., 2020). Over the past 60 years, the number of plastics produced worldwide has risen significantly, from 0.7 million tonnes in 1960 to 348 million tonnes in 2017 (Elgarahy et al., 2021). It is estimated that nearly 300 million tonnes of plastic debris are floating on the ocean’s surface worldwide (Shen et al., 2020). Plastics are widely distributed across various environments, including freshwater, marine ecosystems, land, and air (Prata et al., 2021). Plastic polymers are pervasive and long-lasting, posing a significant threat to aquatic environments (Arienzo et al., 2021). They have been documented as life-threatening substances that cause a wide range of physiological, chemical, and biological toxic effects in animals that mistakenly ingest them as food particles (de Souza et al., 2022).

The abundance of microplastics (MPs) in surface waters is increasing globally and poses a considerable environmental challenge and ecological dilemma. It is estimated that approximately 6.35 billion plastic pieces, weighing 368,840 tonnes, exist worldwide (Geyer et al., 2017). Gasperi et al. (2018) reported the presence of microplastics in surface waters, with concentrations reaching up to 5,000 particles per liter (Zhang et al., 2019). In Pakistan, about 8,000 plasticizers produce 0.8 million tonnes of plastic, contributing 0.6 million tonnes of plastic waste that makes its way into aquatic environments (Dawn, 2019). According to the International Trade Administration, 9% of the entire solid-wastes produced in Pakistan comprises plastics and plastic-products, which are often dumped along the banks of rivers, drains, canals, and freshwater bodies (International Trade Administration, 2019).

Despite the production and overconsumption of plastics, Pakistan lags in assessing the impacts of plastics and plastic debris on aquatic environments, often neglecting the effects of plastic debris in surface waters (Avery-Gomm et al., 2018). Irfan et al. (2020) documented the widespread occurrences of MPs in the river Ravi, with concentrations ranging from approximately 12–35 pieces per cubic meter in different sections. Polyethylene bags and polystyrene pieces were also identified drifting along the water flow. Microplastics in surface waters can act as carriers of other toxicants, resulting in potentially lethal effects when ingested (Wang et al., 2021). Previous literature has documented the occurrence, concentration, and distribution of microplastics in various freshwater ecosystems. Therefore, it is urgently necessary to specify the abundance and concentrations of microplastics in densely populated areas.

Aquatic birds often serve as important biological indicators for aquatic ecosystem. As active predators relying on lower trophic levels for resting, nesting, and feeding, they have a high capacity to acquire microplastics through bioaccumulation and biomagnification, making them significantly impacted by microplastic pollution (Kupekar et al., 2015). Additionally, the diverse habitats, large colonies, and ease of observation make these birds ideal subjects for researchers to collect samples from specific locations (Kramar et al., 2019). Various documented literature has examined the stomach contents of various bird species to better understand microplastics magnification and accumulation. However, previous studies have shown that freshwater birds have comparable levels of microplastics to seabirds (Holland et al., 2016). Recent data estimate that 57% of marine birds and 54% of freshwater birds are affected by microplastics, with projections that by 2050, about 99% of bird species will harbor microplastics in their bodies, affecting their circulatory systems, organs, and respiratory tracts (Wilcox et al., 2015). Therefore, it is mandatory to document the microplastics concentration in locality of boroughing areas. This research work is the primary documentation of its nature, highlighting the abundance of MPs from surface water in vicinity of River Ravi, a major MPs dumping site from district Lahore. The aim of the recent research work was to estimate the ubiquity and measurement of MPs in canals and drains of district Lahore, Pakistan, accentuating the microplastics potential role in population decline of wild birds in vicinity of River Ravi.

## 2 Materials and methods

### 2.1 Experimental animal handling and sampling strategy

**FIGURE 1:**
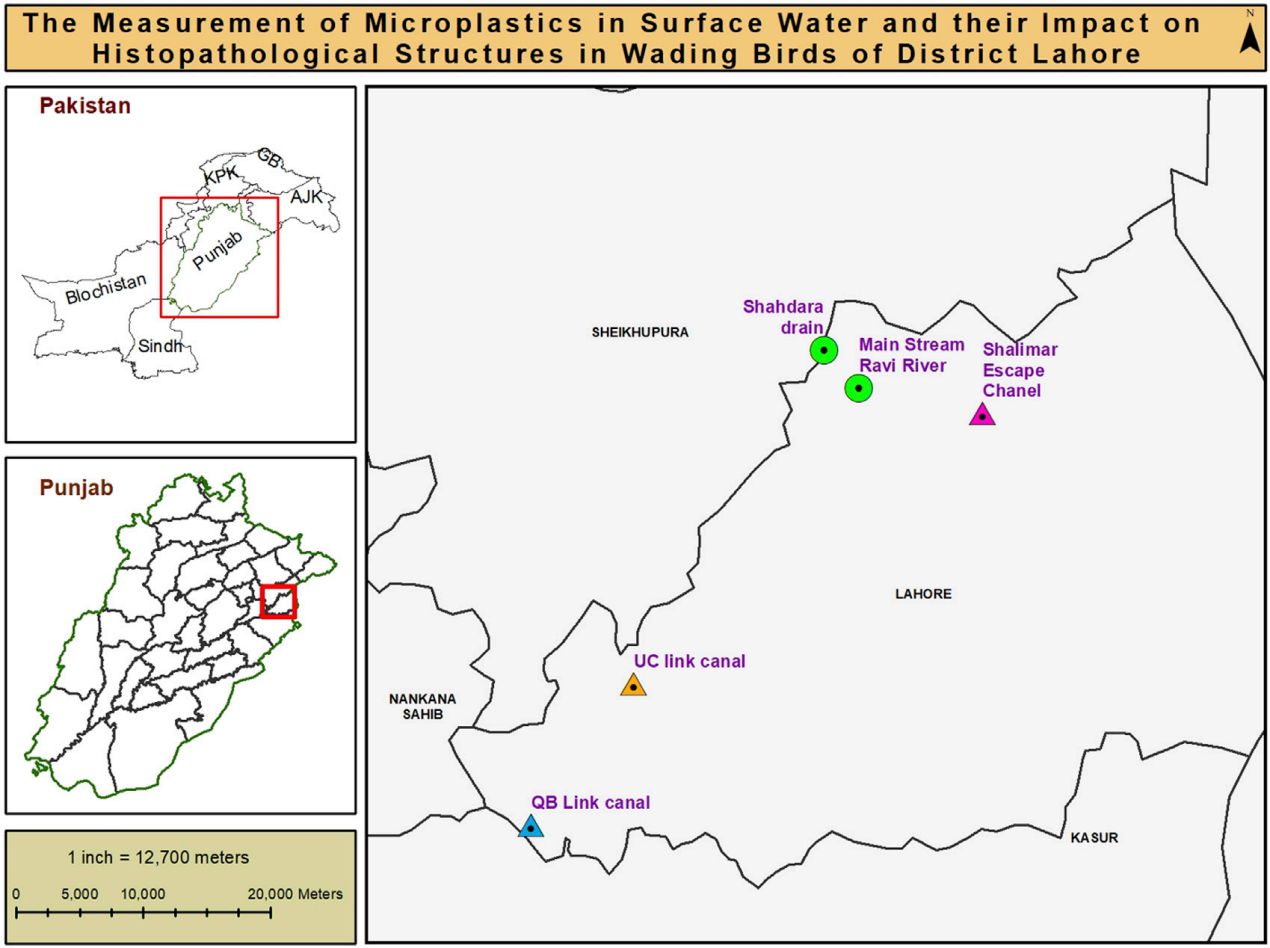
Map showing sampling sites Ravi River, drains, link canals.

In the present study, Red-Wattled Lapwings (*Vanellus indicus*), with 10 samples from each of two different sites (n = 20) the main stream and Shahdara drain were captured using the RSS technique in District Lahore (Figure 1). At each sampling location (n = 10), the selected bird species were caught by placing a mist net (8-m long × 60-cm high). The birds were safely removed from the nets following the procedures described by Spencer et al. (2021), placed into cotton cloth bags separately, and transported to the laboratory of the Department of Wildlife and Ecology, University of Veterinary and Animal Sciences, Lahore, for further processing.

### 2.2 Sample preparation and MPs identification

Water samples were collected from each selected site. Surface water at a depth of 0–1 m was collected in repeatedly washed bottles, transported to the laboratory, and kept at 5°C prior to further investigations (Leslie et al., 2017; Wang et al., 2018). In the laboratory, samples were filtered using sieves of 5 mm and 0.3 mm, respectively. Microplastics (MPs) larger than 1 mm were visually identified. To remove organic matter from the samples, an organic digestion was performed. Specifically, the filtered samples were mixed with 10 mL of H₂O₂ and kept at 20°C–25°C for 24–72 h (Allen et al., 2019). To filter the supernatant, Whatman filter paper with a 0.7 μm pore size was used under vacuum conditions. The filter paper was then placed in a petri dish, dried at room temperature and examined under optical microscope attached to a digital camera. MPs particles were identified according to the protocol prescribed by Hidalgo et al. (2012). Samples were grouped based on the observed sizes of the filtered water samples into Groups: G_1_ (<0.5 mm), G_2_ (0.5–1 mm), G_3_ (1–2 mm), G_4_ (2–3 mm), G_5_ (3–4 mm) and G_6_ (4–5 mm).

### 2.3 Frequency of occurrence of ingested plastic

The sampled birds were necropsied and the ingested plastic items retrieved from organs (ventriculus, digestive tract and crop) were collected, washed, dried, categorized according to their morphology (fibers, fragments, sheets, beads, foam) and size (<5 mm) by following the protocol given by Provencher et al. (2017). The prevalence of ingested plastics in terms of percentage frequency occurrence (%FO) was determined according to the procedures outlined by Lavers et al. (2014). The frequency of occurrence (%FO) was calculated by using formula:

$$\text{FO}\left(\%\right)=\frac{\text{Number}\;\text{of}\;\text{Birds}\;\text{with}\;\text{ingested}\;\text{plastics}\;}{\text{Total}\;\text{number}\;\text{of}\;\text{birds}\;\text{examined}}\;\times 100$$

### 2.4 Sample preparation for tissue digestion

For the tissue analysis, the digestion of tissues, density-based separation of microplastics and detection was evaluated by following the protocol of Li et al. (2018). After dissection, the tissues were carefully placed in pre washed conical flasks, and chemical digestion was done by using 10% potassium hydroxide solution, flasks were wrapped in aluminum foils, dried in oven at 60^O^C for 24-hrs for complete digestion. For density separation the digested mixture was diluted using 500 mL of saturated NaCl, shaken well using glass rod and allowed to settle-down for overnight. After settlement of solution the supernatant was filtered using cellulose-membrane filter paper (47 mm, 0.45 μm), visualized under optical microscope attached with digital camera.

### 2.5 FTIR analysis

Water samples were collected from both sites and submitted to the Interdisciplinary Research Center in Biomedical Materials, COMSATS University Islamabad, Lahore Campus, to analyze the chemical composition of identified MPs using Fourier Transform Infrared Spectroscopy (FTIR) with Bench Serial Number: AUP2010569.

### 2.6 Histology

For histological observation, liver and kidney tissues were extracted, fixed in 10% buffered formalin for about 12 h, and then submitted to the laboratory of the Department of Pathology, University of Veterinary and Animal Sciences, Lahore, for histological observations. The quantitative and qualitative assessment of liver histological-alterations was achieved using the procedures provided by Bernet et al. (1999). Histological alteration indices (HAI), frequency, and prevalence percentages were calculated to compare the intensity of deterioration in bird organs. The associated specific modifications were represented by “w” (an importance factor), indicating the extent of damage. The average histological alterations indices (HAI) were quantified by following the modified protocol of Paulo et al. (2012), determining the extent of damage and severity of lesions in MPs-exposed *V. indicus* species using the following formula:

$$\text{HAI}=\sum_{i}^{n}=1\;\left(a_{i}\times w_{i}\right)$$

a = score value for i-th histological parameter

w = importance factor for i-th histological parameter

n = number of histological parameters assessed.

HAI ≤10 Normal tissue physiology with no observed lesion.

HAI ≤20 Slight tissue modifications and alterations with modest lesions.

HAI ≤50 Moderate tissue alterations with curable lesions.

HAI ≤100 Severe tissue damage with permanent lesions with no possibility to cure.

HAI ≥100 Abnormal organ physiology with marked lesions and tissue damage leading to organ dysfunction-ing with no possibility of organ recovery.

### 2.7 Quality assurance/quality control

To minimize contamination, all equipments were washed with distill water before each use. Laboratory coats and surgical gloves were worn, and metal or glass containers were preferred over plastic ones. These procedures reduced the contact time with air and minimized airflow, thereby preventing fiber contamination. Throughout the entire experimentation process, all samples were protected using aluminum wraps to avoid contamination. All reagents were filtered using Whatman filter paper. Laboratory procedure blanks were set up to monitor contamination during sample analysis, with two blanks sampled—one from each selected site, Shahdara drain and the main stream of the River Ravi. Moreover, an average of 1.0 ± 0.7 plastic particles was observed, indicating that contamination from laboratory and field environmental procedures was negligible.

### 2.8 Data analysis

A one-way analysis of variance (ANOVA) and Pearson correlation was performed using (SPSS) Version 21.0 software (IBM, United States) to investigate the variance and correlation respectively between the concentrations of different types and sizes of MPs. The spatial distribution of MPs was mapped using ArcGIS (Version 10.3), and graphs were prepared using Origin Pro 2016. A one-way analysis of variance and Chi-square test were used to analyze the histological lesions using the Statistical Package for Social Sciences (SPSS) Version 21.0 software (IBM, United States).

## 3 Results

### 3.1 Concentration profile and structural features of MPs in water

**FIGURE 2:**
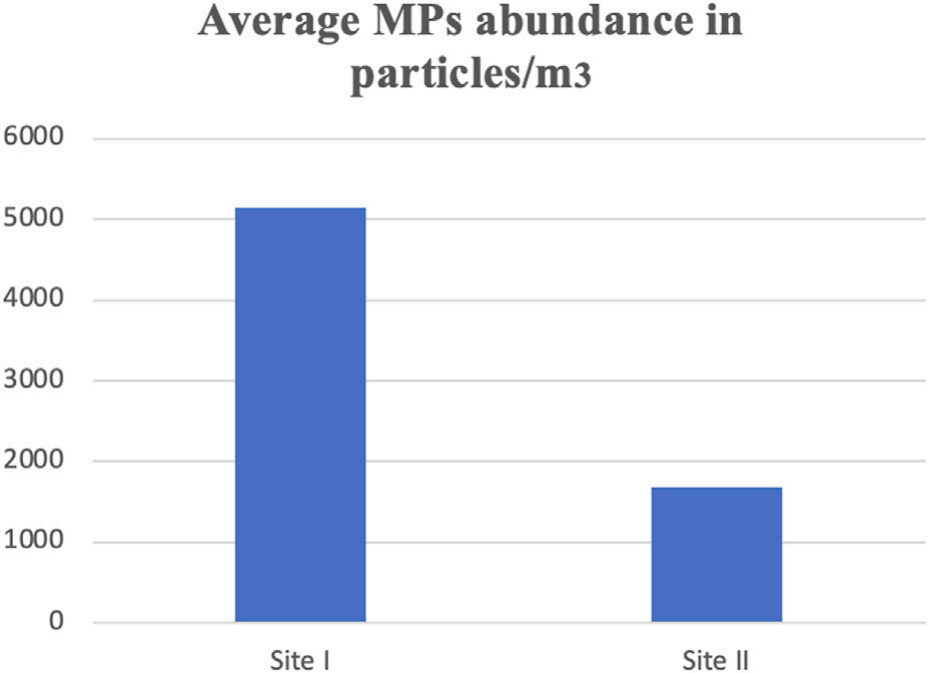
Microplastics concentrations at studied sites.

Microplastic particles from the surface water and targeted tissues of V. indicus were retrieved from the selected locations, namely, Main Stream (Site I) and Shahdara Drain (Site II), and were categorized both qualitatively and quantitatively. The concentration ranged between 5,150 ± 7.5 and 1,673 ± 10.4 particles/m³ (Figure 2). A significant difference in MPs concentration was observed between the selected sites, with the highest mean of 5,150 ± 7.5 particles/m³ in the main stream of the Ravi River (Figure 2). Microplastics abundance (MPs/m³) was calculated by measuring the amount of MPs per volume of filtered water sample (Masura et al., 2015). Five different forms of microplastics—fibers, fragments, foam, beads, and sheets—were observed at both sites. Based on morphological characteristics, fibers were dominant (77%) in the surface water of sampling location I, followed by fragments (19%), sheets (5%), foam (3%), and beads (2%) as shown in (Table 1). Site I showed the highest mean concentration of fibers at 92.4 ± 0.3, whereas the lowest mean concentration of 29.9 ± 0.1was observed at Site II. In contrast, fragments were dominant at Site II, following the order: fragments (35%) > fibers (24%) > sheets (3%) > foam (2%) > beads (1%). The dominance of fibers and fragments at both sites suggests that their origin is secondary. Additionally, the presence of secondary MPs indicates poor waste management strategies at both sites. The factors influencing the abundance of microplastics included densely populated urban centers, open cultivated areas near urban regions, and riverbanks (Klein et al., 2015).

**TABLE 1:** Morphological abundance of microplastics in surface water at studied sites.

	Fibers	Fragments	Sheets	Foam	Beads
Site I	92.4 ± 0.3	21.7 ± 0.1	5.4 ± 0.1	3.5 ± 0.2	2.1 ± 0.3
Percentage (%) Prevalence	77%	19%	5%	3%	2%
Site II	29.9 ± 0.1	42.6 ± 0.3	3.9 ± 0.1	1.9 ± 0.2	1.2 ± 0.1
Percentage (%) Prevalence	24%	35%	3%	2%	1%

**FIGURE 3:**
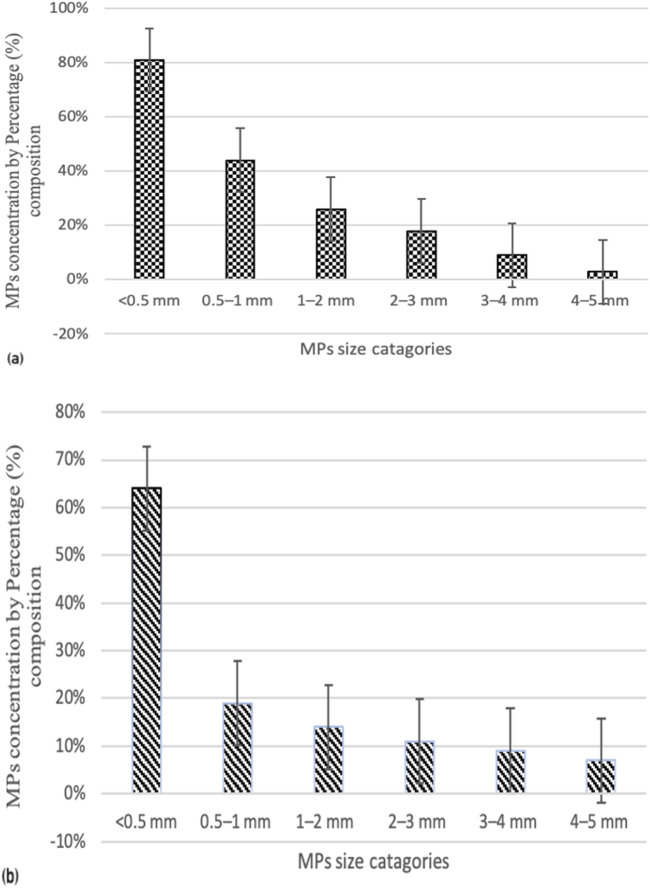
Microplastics concentrations based on size categories at site I **(A)** and site II **(B)**.

In terms of size, large MPs pieces (400 μm-5 mm) were found in the highest quantities in the surface water of both locations (Figure 3). Particles smaller than 0.5 mm were the most prevalent at Site I of the Ravi River, accounting for 56%, with the lowest proportions at Site II following the order: 0.5–1 mm (24%), 1–2 mm (16%), 2–3 mm (8%), 3–4 mm (5%), and 4–5 mm (3%).

### 3.2 Frequency of occurrence of ingested plastic in organ of *V. indicus*

The frequency of occurrence (%FO; prevalence) of plastics in necropsied birds was 89.7% (18/20). Birds from Site I had 100% in their organs containing plastic items compared to those from Site II. This site receives more plastics, with approximately 0.7-billion particles released into the Ravi-River from different sources (drains) within 24-hours. Consequently, birds from this locality exhibited a higher ingestion of plastic items, following the trend: Site I > Site II) Table 2.

**TABLE 2:** Frequency of occurrence of ingested plastic in organs of *V. indicus* at studied sites.

Studies locations	Organs	Fibers	Fragments	Foam	Sheets	Beads
Site I	Ventriculus	40.71	12.83	7.12	0.24	0.21
	Digestive tract	16.40	9.85	1.61	0.22	1.10
	Crop	7.21	4.66	_	1.69	_
	%FO	54%	22%	7%	2%	1%
Site II	Ventriculus	32.74	31.80	1.11	0.25	0.14
	Digestive tract	19.81	27.52	0.73	0.18	1.05
	Crop	0.87	8.92	_	1.51	_
	%FO	44%	57%	1%	1%	1%

Overall, the necropsied birds ingested 95.7% fibers, 5.6% fragments, and less than 1% foam, threads, sheets, and other items. A similar pattern was observed in birds at Site I (fibers: 54%, fragments: 22%, foam: 7%, sheets: 2%, and beads: 1%), while a different concentration was found at Site II (fibers: 44%, fragments: 57%, foam, sheets, and beads: <1%). There was no significant difference in the types of plastics found between the selected sites. A total of 120 items were analyzed: 64 fibers, 23 fragments, 10 pieces of foam, 14 pieces of sheet, and 9 beads. Of the total ingested plastic debris analyzed, the largest proportion was comprised of polyethylene, making up 46% of the samples. Polypropylene was the second most common type of plastic found, representing 27% of the total samples, and was the most common resin type in both nurdles and fragments (66.7% and 62.7%, respectively). Polystyrene was the most common resin type in foam samples (84%). Notably, the most abundant type of plastic found, polypropylene (PP), is currently not recyclable.

### 3.3 FTIR analysis

**FIGURE 4:**
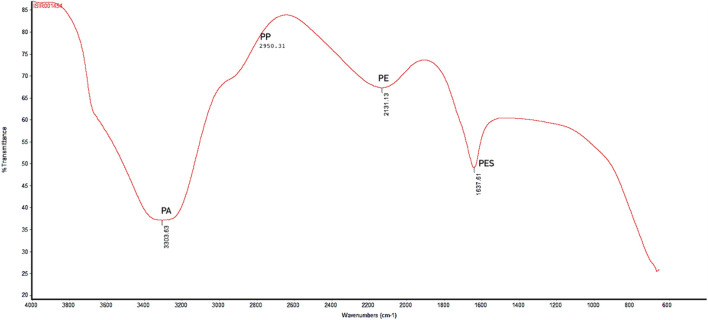
FTIR analysis for polymer characterization in water.

Polymer characterization provides valuable information about the likely sources of plastics, determining whether they originate from primary or secondary sources (Desforges et al., 2014). The FTIR analysis showed the results in spectrum, a plot of transmittance (absorption) against the wavenumber (cm^−1^). The spectrum comprises of different peaks at various wavenumber (cm^−1^). Each peak corresponds to specific bond vibrations in a sample, allowing the identification of sample chemical analysis. *X*-axis of spectrum represents the wavenumber (cm^−1^) against the wavelength of absorbed light in sample. The *y*-axis represents the transmittance (absorption) of light at each wavenumber. Figure 6 showed several distinct peaks, labeled with their corresponding wavenumbers. Different peak values showed different type of polymers present in the water sample. A peak at 2,131.13 cm⁻^1^ spectrum indicates the triple bond functional group. The polymer with triple bond is commonly used in fiber production and are precursors of carbon fibers, belonging to polyethylene type of polymer. Hence, this peak indicates typically presence of polyethylene in the sample. Whereas, peak with 1,637.61 cm⁻^1^ wavenumber is specifically associated with C = C stretching vibrations. The polymers with this type of double bond (C = C) are known as polystyrene. Particularly, the polystyrene (unsaturated polymer) with double bond indicates the spectrum ranging between 1,600 and 1,650 cm⁻^1^ wavenumbers. The spectrum with peak 3,303.63 cm⁻^1^ is typically associated with polyamides forming a single bond between N-H stretching-vibrations. The peak represents the characteristic amide group (amide-linkage) ranging between 3,300 and 3,500 cm⁻^1^. 2,950.31 cm⁻^1^ peak is the representative of polypropylene type of polymer with asymmetric methyl (CH_3_) group vibrations and is regarded as backbone of the polymer. Of the 120 MPs particles identified, 24 were analyzed using FTIR spectroscopy, with 22 revealing their polymer composition. Polyester (PES; n = 5), polyethylene (PE; n = 9), polypropylene (PP; n = 6), and polyamides (PA; n = 2) were detected (Figure 4).

### 3.4 Clustered heatmap and correlation analysis

**FIGURE 5:**
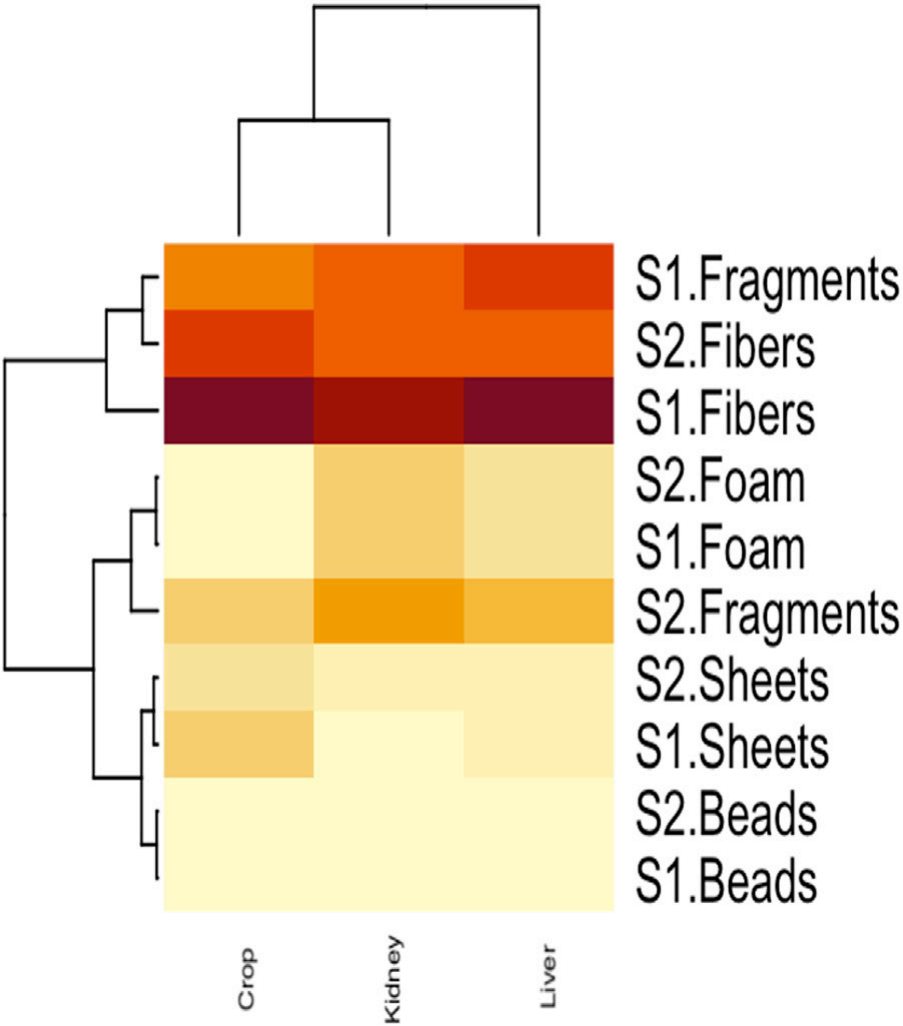
Clustered heatmap analysis representing the relationship between MPs morphotype and their distribution in *V.indicus* effected organs*.*

**FIGURE 6:**
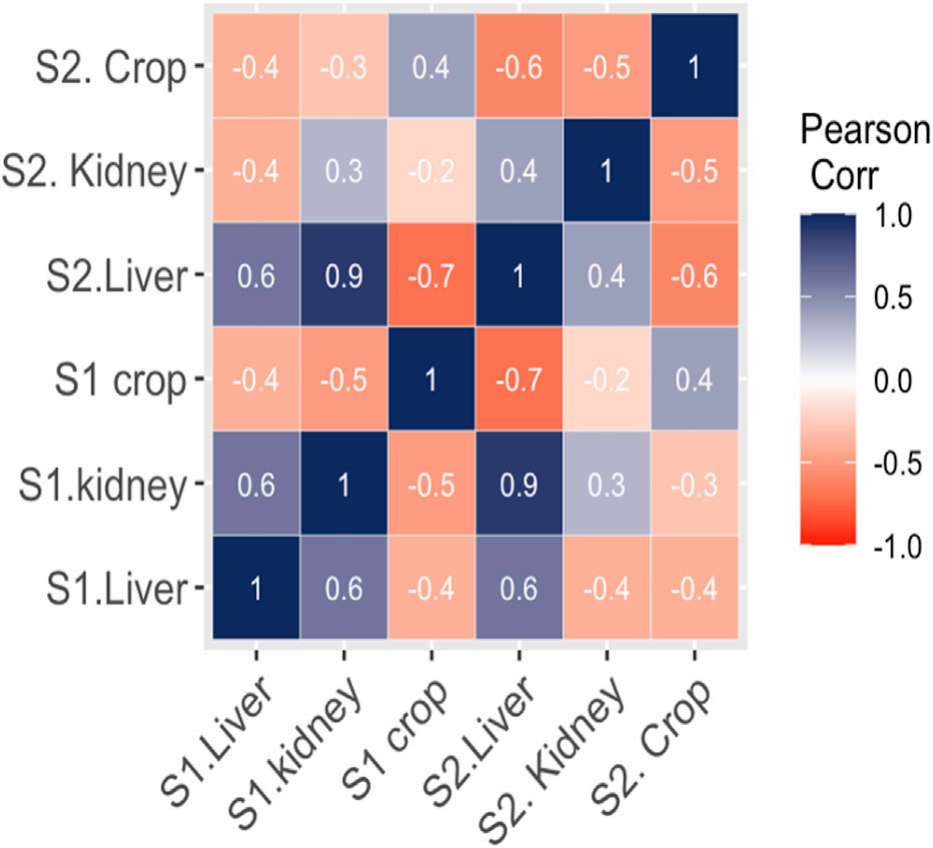
Pearson correlation (*p* > 0.05) between MPs morphotype nd *V.indicus* effected organs.

The interaction between MPs morphotypes, the model species, and their impacts on targeted organs were summarized using clustered heatmap analysis from both studied sites (I and II). A significant (p ≤ 0.05) association was found between microplastic morphotypes and the affected organs of V. indicus. The kidney, liver, and crop exhibited a higher proportion of fibers from both studied sites. However, damage caused by MPs fragments were more prevalent in the organs (liver, kidney, and crop) from Site II compared to Site I. The proportion of MPs in the crop did not show a significant association. The major portion of MPs in the liver and kidney consisted of fibers, following the order: fibers > fragments > sheets. The liver and crop from Site I showed a strong association with fiber morphotypes, whereas the kidney from Site I showed a weaker association with fibers. Additionally, the crop from Site II showed a strong negative association with sheet and bead morphotypes and a weak negative relationship with fragments. The kidney showed a strong positive association with foam from both Site I and Site II, a weak negative association with sheets, and a strong negative association with beads (Figure 5). Pearson correlation was evaluated among MP abundance and the affected organs of V. indicus. The results showed that microplastic abundance in the bird’s targeted organs was significantly corelated(p > 0.05). A strong positive relationship was found between the liver and kidney as compare to the crop of bird-species sampled from both sites (Figure 6).

### 3.5 Microplastics morphotype

**FIGURE 7:**
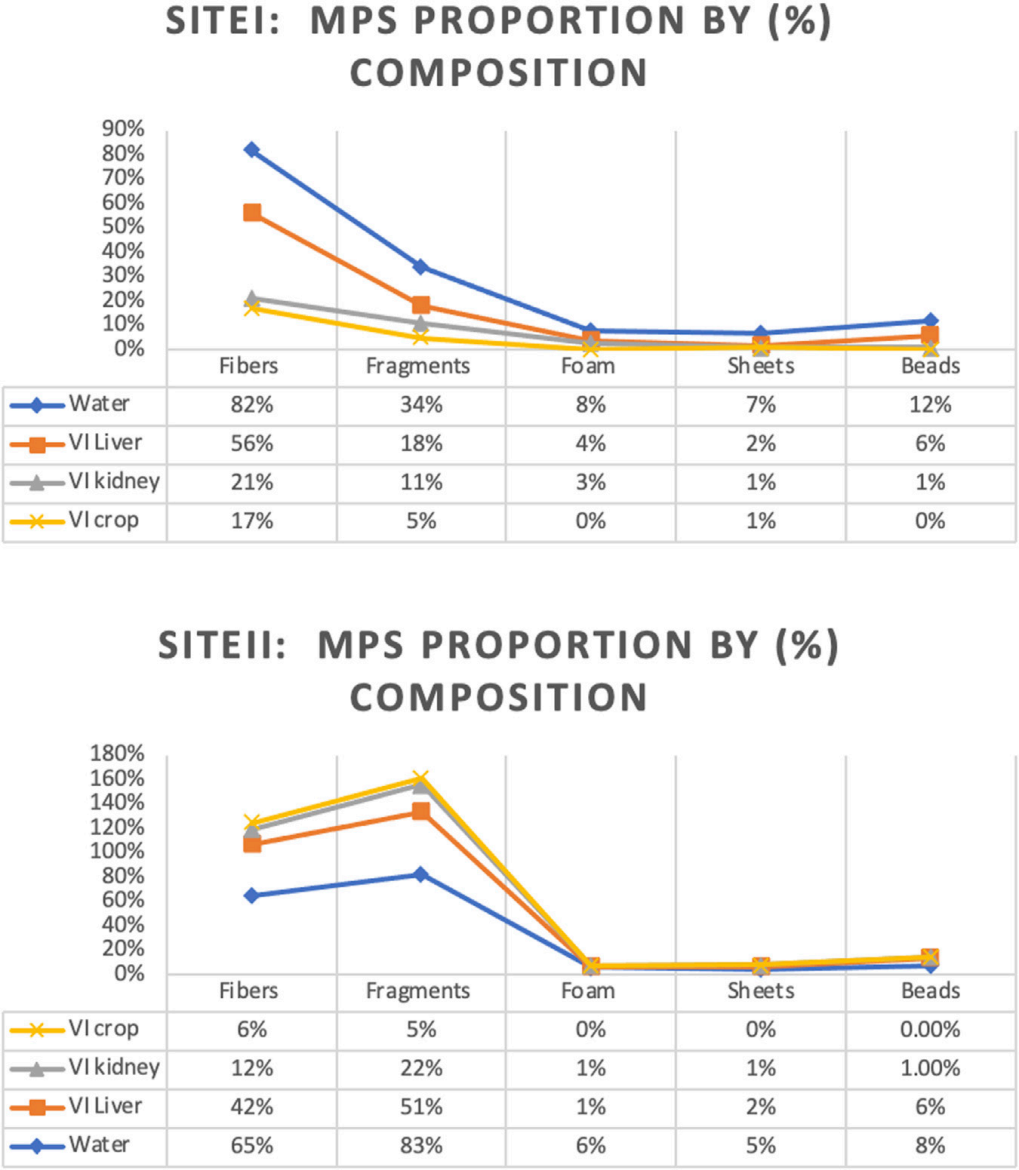
MPs percentage composition in water and *V.indicus* organs.

Various morphotypes of MPs were identified from water and bird organs at both sites in the vicinity of the Ravi River (Figure 7). The results indicated that MPs were categorized based on their shapes as fibers, fragments, sheets, foam, and beads. The abundance of these shapes followed the order: fibers > fragments > sheets > foam > beads in water samples from Site I. However, sheets were not observed in any of the bird tissues, and fragments were the most abundant at Site II, following the order: fragments > fibers > sheets > foam > beads.

### 3.6 Histopathological changes in *V. indicus*

**FIGURE 8:**
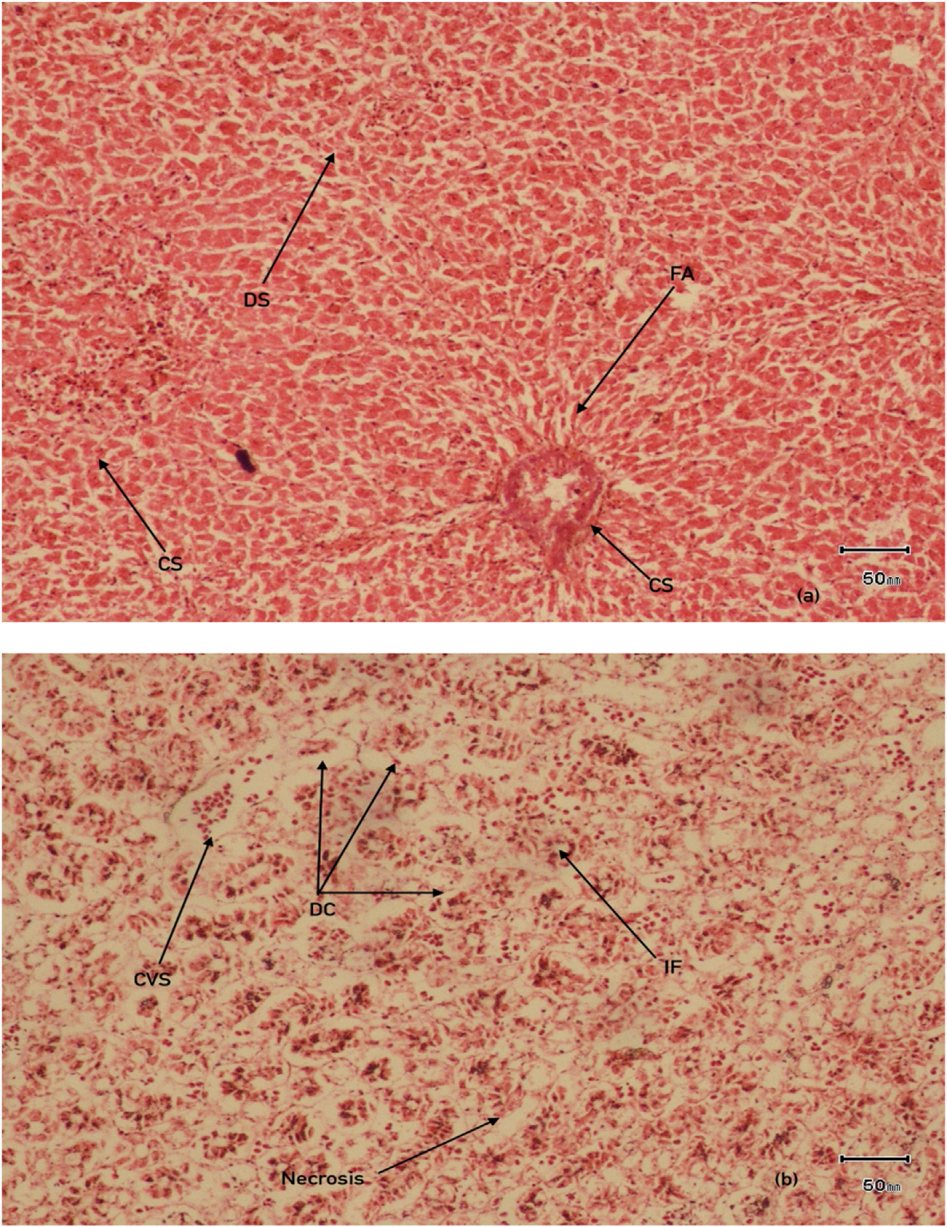
Histological examination of organs **(A)** Liver **(B)** kidney in MPs exposed *V.indicus* IF, infiltration of inflammatory cells DS, dillation of sinusoids CS, congestion of sinusoids, DC, degeneration of renal epithelial cells, CVS, central vein congestion, FA, focal area of necrosis.

Histological observations revealed that many hepatocyte nuclei were pyknotic, and mild infiltration of inflammatory cells was observed in the portal area. Marked degeneration and necrosis were noted in the majority of hepatocytes within the hepatic cords of the liver in V. indicus from both sites. Additionally, necrosis of renal epithelial cells was evident, with most cells sloughed off and deposited in the lumen, although the basement membranes remained intact in the kidney (Figure 8). The histological parameters, including frequency and prevalence percentage, were significantly higher in birds exposed to MPs. The highest prevalence percentage, 90%, was observed in the liver as degeneration of hepatocytes in 0.5–1 mm and pyknotic nuclei in 0.5 mm-sized MPs. The lowest prevalence percentage, 5%, was observed in 4-5 mm-sized MPs (Table 3). The frequency and prevalence percentages followed the order: 0.5 mm > 0.5–1 mm > 1–2 mm > 2–3 mm > 3–4 mm > 4–5 mm > 0 mm (0 mm as control). In kidney tissues, the highest prevalence percentage, 85%, was observed as necrosis in renal tubular epithelial cells, with the lowest prevalence percentage, 30%, observed as hyperaemia in 0.5 mm-sized MPs-exposed bird species (Tables 3, 4).

**TABLE 3:** Frequency, prevalence percentage (%) and histological alteration indices (HAI) values observed in liver of MPs exposed bird species.

Histological alterations	Frequency							Percentage (%)						
	0.5 mm	0.5–1 mm	1–2 mm	2–3 mm	3–4 mm	4–5 mm	0 mm	0.5 mm	0.5–1 mm	1–2 mm	2–3 mm	3–4 mm	4–5 mm	0 mm
Haemorrhage	15	10	8	4	7	5	0	75	50	40	20	35	25	0
Focal area of necrosis	16	16	15	9	3	5	0	80	80	75	45	15	25	0
degeneration of hepatocytes	17	18	11	8	5	2	0	85	90	55	40	25	10	0
Pyknotic nuclei	18	10	17	12	8	2	0	90	50	85	60	40	10	0
Hepatic necrosis	14	11	9	7	7	3	0	70	55	45	35	35	15	0
Congestion of sinusoids	13	12	9	8	5	1	0	65	60	45	40	25	5	0
Dilation of sinusoids	9	6	4	1	2	4	0	45	30	20	5	10	20	0
Inflammation	12	17	11	8	6	9	0	60	85	55	40	30	45	0
Liver index, I_L_														

Importance factor (w)	Index (I)	Score value (a)							HAI value (a×w)						
		0.5 mm	0.5–1 mm	1–2 mm	2–3 mm	3–4 mm	4–5 mm	0 mm	0.5 mm	0.5–1 mm	1–2 mm	2–3 mm	3–4 mm	4–5 mm	0 mm
wLC1 = 2	ILC	4	3	2	1	2	2	0	8	6	4	2	4	4	0
wLR1 = 3	ILR	6	2	2	2	1	2	0	18	6	6	6	3	6	0
wLR2 = 3	ILR	6	2	2	2	2	1	0	18	6	6	6	6	3	0
wLR2 = 3	ILR	6	3	1	1	2	2	0	18	9	3	3	6	6	0
wLR2 = 2	ILR	4	2	1	2	1	2	0	8	4	2	4	2	4	0
wLP1 = 1	ILP	2	1	1	1	1	1	0	2	1	1	1	1	1	0
wLP2 = 1	ILP	2	1	1	1	1	1	0	2	1	1	1	1	1	0
wLI1 = 3	ILI	6	4	4	2	2	2	0	18	12	12	6	6	6	0
									92	45	35	29	29	31	0

**TABLE 4:** Frequency, prevalence percentage (%) and histological alteration indices (HAI) values observed in kidney of MPs exposed bird species.

Histological alterations	Frequency							Percentage (%)						
	0.5 mm	0.5–1 mm	1–2 mm	2–3 mm	3–4 mm	4–5 mm	0 mm	0.5 mm	0.5–1 mm	1–2 mm	2–3 mm	3–4 mm	4–5 mm	0 mm
Hyperaemia	6	11	9	4	1	5	0	30	55	45	20	5	25	0
Necrosis	18	11	7	9	2	7	0	90	55	35	45	10	35	0
Renal tubular epithelial cell degeneration	16	12	7	4	8	1	0	80	60	35	20	40	5	0
Cellular sloughing	9	6	4	7	9	3	0	45	30	20	35	45	15	0
Cellular swelling in epithelial cells	14	8	9	5	7	4	0	70	40	45	25	35	20	0
Kidney index, IK														

Importance factor (w)	Index (I)	Score value (a)							HAI value (a×w)						
		0.5 mm	0.5–1 mm	1–2 mm	2–3 mm	3–4 mm	4–5 mm	0 mm	0.5 mm	0.5–1 mm	1–2 mm	2–3 mm	3–4 mm	4–5 mm	0 mm
wKC1 = 1	ILC	6	3	2	5	2	1	0	6	3	2	5	2	1	0
wKR1 = 3	ILR	6	2	4	4	3	2	0	18	6	12	12	9	6	0
wKR2 = 3	ILR	4	4	2	2	1	1	0	12	12	6	6	3	3	0
wKR3 = 2	ILR	4	3	1	4	2	3	0	8	6	2	8	4	6	0
wKP1 = 3	ILP	6	2	2	6	4	2	0	18	6	6	18	12	6	0
									62	33	28	49	30	22	0

ILC, index of liver circulatory changes; ILR, index of liver regressive changes; ILP, index of liver progressive changes; ILI, index of liver inflammatory changes, w importance factor, a score value, HAI, histological alteration index; IL, liver indices.

IKC, index of kidney circulatory changes; IKR, index of kidney regressive changes; IKP, index of kidney progressive changes, w importance factor, a score value, HAI, histological alteration index; IK, kidney indices.

The tissue repair capacity was categorized into three progressive stages: I - recovery of damaged tissue to normal physiology is possible; II - no recovery of damaged tissue, affecting normal physiology; III - permanent damage to tissue with severe damage to organ function, leading to death. The current results regarding the severity of lesions and the significant extent of organ (liver and kidney) damage and alterations were assessed by computing HAI values. The mean HAI value for the liver was 92, while the degree of damage was 61%, falling into the category of HAI ≤100, representing stage III with severe lesions and alterations in tissue structure and function, with a 0% possibility of recovery. This indicates that MP-induced damage to the bird species could potentially cause modifications in tissue and organ vitality, inducing a stressed condition that makes bird species vulnerable to disease. The highest HAI indices, 92 for the liver and 62 for the kidney, were observed in 0.5 mm-sized MPs (Tables 3, 4).

## 4 Discussion

### 4.1 Microplastics abundance in water and organs of *V. indicus*

MPs pollution is emerging as a significant global issue, but information about their abundance, dissemination, and associations with freshwater systems is still developing. Therefore, the current study examined the impacts of microplastics on aquatic fauna in District Lahore as a model species to evaluate the potential health consequences for humans. This study is the first in the literature to evaluate the impacts of microplastics on wild fauna from polluted sites in District Lahore. Moreover, the studied locations are considered among the most microplastic-polluted sections, where the majority of wastewater is disposed of from various sources into the freshwater River Ravi. According to Sabiha et al. (2015), approximately 1,500 tonns of solid wastes is produced daily, and a Town Municipal Authority (TMA) survey documented that 18.8% of this waste consists of plastics and plastic particles. Therefore, it is believed that household, municipal, and industrial sewage may be the reason for the high MP concentrations documented in the present study. Similar results were reported by Auta et al. (2017), who identified municipal sewage as a significant source of MP pollution, produced from the degradation of large plastics.

The present study documented fibers (54%) as the dominant morphotype at Site I. Fibers as a dominant polymer have been frequently reported (Li et al., 2019). Moreover, the polymer composition reported here is comparable to previous studies (Shahul Hamid et al., 2018). Fibers (44%) were not exceptionally abundant compared to fragments (57%), which were more prevalent in surface water at Site II followed by beads, sheets, and foam (<1%). The abundance of fragments might be due to their abundance, as they are originated from the degradation of large plastics and plastic pieces, including various types of plastic containers, tin packs, and packaged items (Cai et al., 2020).

Our findings concerning the abundance of fibers (82%) in water (Figure 8) are comparable with previous studies, such as Yuan et al. (2019), which documented the highest fiber percentage (42%) in water. Similar data were found in Taihu Lake, China, with 48%–85% (Su et al., 2016), Hong Lake with 44.3%–84.1%, and Dongting Lake with 42%–92% (Wang et al., 2018). Microplastics were present in all sampled species, representing the significant impacts of water pollution, which are comparable to previous studies by Roman et al. (2019). They found that interactions between wildlife and microplastics directly contribute to mortality aa s potential source of decline in the diversity of aquatic fauna. Additionally, another study showed that MPs are a significant component of the digestive tract of many aquatic animals (Carlin et al., 2020). A similar investigation by Kuhn and van Franker (2020) revealed that the ingestion of deposited and aggregated MPs or larger plastic debris can result in bleeding, blockage of the presented digestive tract, ulcers, or perforations of the gut, producing a deceptive sense of satiation, leading to starvation or mortality. Currently, half of the world’s seabird species are expected to be affected in this way. Yuan et al. (2019) documented the impacts of ingested MPs in aquatic organisms, compromising respiratory, digestive, and immune health.

The chemical analysis showed that fibers, beads, and fragments belong to the class polyethylene (Godoy et al., 2019). Foams were composed of polyesters, and the sheets belonged to the class of polypropylene (Geyer et al., 2017). Moreover, the present investigation showed that PE (46%) and PP (27%) were the dominant polymer types at all studied locations, indicating the widespread use of these polymers in the respective sites, including plastic bags, foam boxes, plastic containers, sheets, pellets, packaging goods, and the textile industry (PlasticsEurope, 2016). The abundance of polyethylene (PE) in water was consistent with previous literature (Zhao et al., 2019).

### 4.2 Histology

#### 4.2.1 Liver

Interpretative histopathological sections of the liver and kidney of V. indicus is given in Figure 8. The liver, a vital organ involved in the detoxification of pollutants (Amereh et al., 2020), is more prone to MP accumulation as it is consistently exposed to pollutants more than other organs. Most of these effects have not yet been reported in aquatic species (Ragusa et al., 2021). Previously documented literature from some laboratory experiments has revealed that MPs ingestion may results in anatomical and morphological alterations in liver tissues, including hydrophobic vacuolization, passive blood congestion accompanied by lesions, inflammatory reactions, and fat accumulation (Jabeen et al., 2018). Moreover, sinusoidal capillary congestion with hemorrhages was common in bird livers at both sites, similar to laboratory investigations of MP toxicity in *Oreochromis niloticus* (Hamed et al., 2021).

In the current study, drastic and marked variations were noticed in the livers of bird species, including pyknosis, mild infiltration of inflammatory cells in the portal area, and degeneration and necrosis in the majority of hepatocytes within hepatic cords. Karyopyknosis is generally associated with necrosis, consisting of karyorrhexis and cytoplasmic hypereosinophilia (Wolf and Wheeler, 2018). Similar findings were documented in laboratory experiments on tilapia exposed to diN-butylphthalate (10 mg/L) (Erkmen et al., 2017). The significant hepatocyte degeneration and vacuolization were comparable to previous experiments on *Danio rerio* exposed to di-butylphthalate (Xu et al., 2015). Inflammation and inflammatory reactions were observed in *Danio rerio* exposed to polystyrene particles of sizes 25 mm and 75 nm (Lu et al., 2016). In addition, severe tissue damage was observed in juvenile cat fish (*Clarias gariepinus*) fed with poly-ethylene particles (Karami et al., 2016b) is comparable to our study findings.

The results, showing significant tissue damage and marked alterations in liver physiology, were potentially severe enough to cause bird death, compared to the negligible HAI values in (control) MPs. These findings align with those of Paulo et al. (2012), Sirimongkolvorakul et al. (2012), and Georgieva et al. (2014), who investigated histological alterations in various fish tissues and computed HAI values to establish the degree of tissue damage. Santos et al. (2014) documented the HAI (histological alteration index) in Sardinella sp. and C. undecimalis from Jansen, Brazil, and assessed that the mean HAI values were 31.8 and 22.2, respectively. The mean histological alteration index values for both fish species fell into the category of HAI ≤50, classified as moderate to severe damage with no recovery, ultimately leading to the death of the fish species.

#### 4.2.2 Kidney

Oxidative stress is the most significant reason of kidney injury, produced due to inhibition of the antioxidant system and overproduction of reactive oxygen species (ROS) (Gur et al., 2022). Previously published studies have shown that contaminant-induced oxidative stress interferes with ROS production, leading to autophagy, fibrosis, and apoptosis, which cause major tissue injuries and damage (Zheng et al., 2022). Mattsson et al. (2017) reported results consistent with the present study, documenting that microplastic ingestion is directly related to tissue damage, leading to fibrosis and degeneration. Hsieh et al. (2021) also supported these findings, as their laboratory experiments on MPs-exposed organisms documented severity, scarcity, and tissue damage. Severe tissue damage naturally induces scar formation, and excessive tissue scarcity leads to the development of pathological conditions known as fibrosis, contributing to organ failure (Wynn and Ramalingam, 2012). Similar results have been observed in wild birds (Rivers et al., 2023), with previous studies reporting that MPs have the potential to induce severe tissue scarring, leading to inflammation and fibrotic organ changes (Shen et al., 2020). While MPs-induced severity, scarcity, and fibrosis have not been extensively investigated in wild organisms ingesting MPs, the current study results will provide a future baseline and help to better understand the impacts of ingested MPs on the organ health of wild animals.

## 5 Conclusion

The current study anticipated data on the presence and abundance of microplastics in surface water of Ravi River. The MPs were in higher proportion in the surface water at studies locations. Moreover, the freshwater bird species also showed MPs accumulation, which is the major issue for all the aquatic species. The current study documented that the microplastics ingestion causes the severe health disturbances, with potential to induce the severe damage in the different organs of wild birds. Due to the potential impacts of plastic on the health of wildlife, our results thus highlight the urgent need to continue to strengthen our knowledge of the sub-lethal impacts of this diverse pollutant. Hence, it is suggested that there should be the inauguration of WWTP to overcome this plastic pollution in Lahore. The manifestation of legislations and policies are the effective ways to control this growing plastic pollution, thus providing the better and protective environment to the aquatic fauna and ecosystem.

## Data Availability

The raw data supporting the conclusions of this article will be made available by the authors, without undue reservation.
